# Enhancing healthcare recommendation: transfer learning in deep convolutional neural networks for Alzheimer disease detection

**DOI:** 10.3389/fmed.2024.1445325

**Published:** 2024-09-20

**Authors:** Purushottam Kumar Pandey, Jyoti Pruthi, Saeed Alzahrani, Anshul Verma, Benazeer Zohra

**Affiliations:** ^1^Department of Computer Science, Manav Rachna University, Faridabad, Haryana, India; ^2^Management Information System Department, College of Business Administration, King Saud University, Riyadh, Saudi Arabia; ^3^Department of Computer Science, Banaras Hindu University, Varanasi, India; ^4^Department of Anatomy, School of Medical Sciences & Research, Sharda University, Greater Noida, Uttar Pradesh, India; ^5^Department of Anatomy, Noida International Institute of Medical Sciences, Noida International University, Greater Noida, Uttar Pradesh, India

**Keywords:** deep learning, Densenet, EfficientNet-B0, Resnet, skull stripping, healthcare, clustering, decision making

## Abstract

Neurodegenerative disorders such as Alzheimer’s Disease (AD) and Mild Cognitive Impairment (MCI) significantly impact brain function and cognition. Advanced neuroimaging techniques, particularly Magnetic Resonance Imaging (MRI), play a crucial role in diagnosing these conditions by detecting structural abnormalities. This study leverages the ADNI and OASIS datasets, renowned for their extensive MRI data, to develop effective models for detecting AD and MCI. The research conducted three sets of tests, comparing multiple groups: multi-class classification (AD vs. Cognitively Normal (CN) vs. MCI), binary classification (AD vs. CN, and MCI vs. CN), to evaluate the performance of models trained on ADNI and OASIS datasets. Key preprocessing techniques such as Gaussian filtering, contrast enhancement, and resizing were applied to both datasets. Additionally, skull stripping using U-Net was utilized to extract features by removing the skull. Several prominent deep learning architectures including DenseNet-201, EfficientNet-B0, ResNet-50, ResNet-101, and ResNet-152 were investigated to identify subtle patterns associated with AD and MCI. Transfer learning techniques were employed to enhance model performance, leveraging pre-trained datasets for improved Alzheimer’s MCI detection. ResNet-101 exhibited superior performance compared to other models, achieving 98.21% accuracy on the ADNI dataset and 97.45% accuracy on the OASIS dataset in multi-class classification tasks encompassing AD, CN, and MCI. It also performed well in binary classification tasks distinguishing AD from CN. ResNet-152 excelled particularly in binary classification between MCI and CN on the OASIS dataset. These findings underscore the utility of deep learning models in accurately identifying and distinguishing neurodegenerative diseases, showcasing their potential for enhancing clinical diagnosis and treatment monitoring.

## 1 Introduction

Neurodegenerative illnesses like AD affect brain cognitive function. It is one of the most common cause of Dementia. The exact cause of disease is still not fully discovered and so the cure. It is believe that it happens due to a combination of genetic, environmental, and lifestyle factors. The protein accumulation in the brain which is Amyloid Plaques is the main cause. The plaques accumulates between the neurons because of which the death of neuron starts. Inflammation in the brain and oxidative damage to neurons are also believed to play roles in the development and progression of Alzheimer’s disease. These processes can further contribute to neuronal dysfunction and death. These disorders cause problems with brain function and impair cognition (1). Progressive decline in cognitive function, including memory loss and diminished cognitive ability, characterizes AD, the most prevalent form of dementia. Conversely, MCI is a transitional stage between typical cognitive aging and AD, distinguished by observable deterioration in cognitive functions that do not significantly impede routine tasks (2). These conditions impose a burden on healthcare organizations as well as society at large, in addition to endangering the health and safety of those affected.

The efficient detection of AD and MCI has become a crucial area of interest in medical research. The progress in neuroimaging methods, including magnetic resonance imaging (MRI), has improved the ability to diagnose these conditions. MRI scans are used to diagnose Alzheimer’s and MCI by examining structural abnormalities, which often require advanced image processing to increase clarity and extract relevant features (3).

The ADNI (4) and OASIS (5) datasets are renowned for their efficacy in diagnosing Alzheimer’s and MCI, both used in this analysis and recognized for their vast human macroscopic MRI data. These datasets cover healthy and AD/MCI patients. MRI images from both datasets are used to identify anatomical changes connected to neurodegenerative illnesses, such as brain volume and cortical thickness (4, 5).

Multiple methods are utilized to preprocess MRI data to increase AD and MCI diagnosis accuracy and comprehension. A Gaussian filter reduces noise and decreases artifacts and electrical noise to improve visual clarity (6), contrast-limited responsive Histogram Equalization (CLAHE) enhances contrast, the image is resized to 224×224 pixels for consistency (7), and CNN model compatibility and intensity levels are normalized across scans (8). Skull stripping eliminates non-brain tissues to focus further investigations on the importance of brain regions, and then Tissue segmentation segments the brain into gray matter, white matter, and cerebrospinal fluid, providing more precise data for study (9). In ADNI additional preprocessing, we performed skull stripping using U-Net (10) to remove the cranium. The brain is cut cross-sectionally along three axes—axial, coronal, and sagittal. The slices are evaluated for quality, and three are selected to show the most essential MRI imaging areas while reducing noise (9, 11, 12).

Many research’s have been working in the area however the work done so far has limitation that this paper is trying to address. The work done by researcher’s is focused on one dataset, where as we have used the multiclass dataset for the research. The improvised preprocessing model that can work on MRI from different datasets and the prediction model provides the consistency accuracy while predicting.

This investigation offers several substantial improvements to the existing Research on the identification of Alzheimer’s and MCI:

- To extract and preprocess the renowned datasets, ADNI and OASIS, from the neuroimaging discipline for the investigation.
- To propose a framework model for early detection of AD using different deep learning techniques such as DenseNet-201, EfficientNet-B0, ResNet-50, ResNet-101, and ResNet-152 for the classification of MCI detection
- To evaluate and analyze the performance of prominent deep learning using performance metrics for making recommendations in healthcare organizations.

The complexity of MRI images for AD and MCI identification highlights cutting-edge deep learning processes. This work contributes to neuroimaging studies and AD/MCI diagnosis as the discipline progresses.

The paper not only addresses binary classification but also emphasizes multiclass classification. The predictive model extends beyond determining whether a subject has AD or not; it also predicts the stage of the disease, such as AD, CN, or MCI.

In binary classification, the model’s output provides a straightforward yes or no answer regarding the presence of AD or another condition. However, in multiclass classification, the model distinguishes between different stages of the disease, offering a more nuanced understanding of the individual’s cognitive health status. This approach is crucial for clinical applications as it allows healthcare providers to not only diagnose the presence of AD but also to categorize the severity or progression of the disease. Such detailed predictions can significantly aid in early intervention, personalized treatment planning, and monitoring of disease progression over time.

The paper is structured around the materials and methods outlined in Section 3, encompassing preprocessing techniques, transfer learning, and notable CNN architectures. Section 4 presents the dataset details and outcomes of the proposed approach. Lastly, Section 5 encapsulates the conclusion and outlines future avenues for the model’s development.

## 2 Literature review

The analysis of the research done so far is represented in this section.

Modern deep-learning architectures are used to identify subtle patterns from the datasets to create powerful AD and MCI detection applications/models. These architectures ensure and advance neurodegenerative condition research. Most prominent advanced deep-learning architectures such as DenseNet-201 (13), EfficientNet-B0 (14), ResNet-50, ResNet-101, and ResNet-152 (15) have been investigated to develop efficient models for detecting Alzheimer’s and MCI. The architectures often extract detailed patterns from complicated datasets and are used with transfer learning.

The DenseNet-201 design operates by establishing dense connections between each layer and all subsequent layers in a feed-forward manner to recycle features efficiently. The connection mentioned above improves the transmission of features and promotes the reuse of features, resulting in more effective use of parameters (13). EfficientNet-B0 prioritizes enhancing model efficiency by scaling the network in many dimensions (depth, breadth, and resolution) to achieve an optimal trade-off (14). ResNet-50, ResNet-101, and ResNet-152 belong to the ResNet (Residual Network) family. This network family includes skip connections, also known as shortcuts, which enable bypassing one or more layers. This technique helps to address the vanishing gradient issue and facilitates the training of intense networks. These skip connections further enhance the propagation of gradients during backpropagation, enabling the model to learn more efficiently (15). Each of these designs offers distinct methods for extracting features and optimizing parameters, making them suitable for various elements of Alzheimer’s MCI detection in MRI datasets.

A 3D-CNN model was trained using ADNI MRI data to distinguish AD from CN. An AD brain mask was found using a genetic algorithm-based Occlusion Map technique, and Backpropagation-based explain ability methods. The recommended model had 87% accuracy in 5-fold cross-validation, mirroring prior findings, whereas an updated 3D-CNN model with 29 brain regions achieved a high validation accuracy using the lrp_z_plus_fast explain ability technique (16). The assessment process exploits shallow CNN architecture on 2D T1-weighted MR brain images. This pipeline rapidly and accurately identifies normal, MCI, and AD. The technique is labeled MCI prodromal AD. They tested it against DenseNet121, ResNet50, and EfficientNetB7 (17). A unique ensemble deep-learning AD classification technique was developed. Soft-NMS consolidates candidate data and improves detection in the Faster R- CNN architecture. Enhanced ResNet50 extracts more complicated visual data. For sequence data processing, the feature extraction network employed Bi-GRU. Improved Faster R–CNN did the classification well (18). Researchers created EfficientNetB2 for AD, MCI, and NC. Front-end Global Attention Mechanism (GAM) in EfficientNetB2 took crucial features. Coordination Attention helped get channel and location data from two-dimensional slice data for appropriate diagnosis. Micro-designing using the ConvNeXt network reduced model complexity and improved categorization. The recommended method outperformed CNNs on AD/NC, AD/MCI, and MCI/NC dichotomous data (19). Investigators created an integrated automated method for guided machine learning-driven selection using K-Means++. A sophisticated deep learning framework using EfficientNetV2S transfer learning and learned features. Trials utilized ADNI and OASIS benchmark datasets. In research and validation, the integrated design outperformed all other models. Model validation was 20-fold. On the ADNI dataset, CN showed 83.64% accuracy against AD, 82.69% against MCI, 71.40% against MCI, and 91.54% on the OASIS dataset (20).

## 3 Materials and methods

The research approach used in this study centers on utilizing the ADNI and OASIS datasets, which are well-known for their extensive human macroscopic MRI data. These datasets include people who are in good health as well as those who have been diagnosed with AD and MCI (4, 5).

### 3.1 Methodology

**Figure 1:**
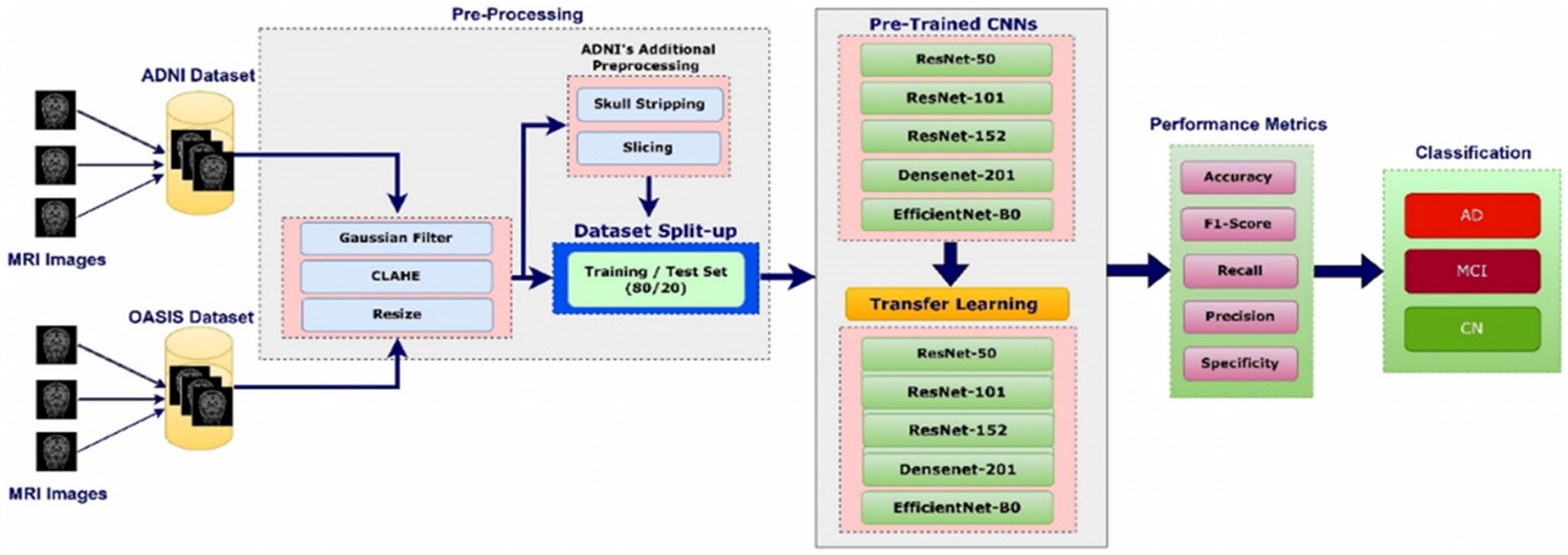
Methodology for the AD and MCI detection.

MRI data is preprocessed using Gaussian filters (6), CLAHE for contrast enhancement, standardized image dimensions, and normalizing intensity levels (7, 8). U-Net removes the cranium for the ADNI dataset, and the brain is sliced along three axes for cross-sectional slices. These slices undergo a quality evaluation to provide the best depiction while minimizing noise and highlighting significant regions of MRI imaging (9–13). State-of-the-art deep-learning architectures like DenseNet-201, EfficientNet-B0, ResNet-50, ResNet-101, and ResNet-152 extract intricate patterns from the datasets, hence aiding in creating effective models for AD and MCI identification. This mechanism guarantees the strength and dependability of the analysis performed on the ADNI and OASIS datasets, enabling progress in comprehending and identifying neurodegenerative disorders (13–15). Figure 1 presents the methodology for detecting AD and MCI using ADNI and OASIS datasets.

During the implementation the steps followed will be explained in the paragraph. The dataset will be provided to preprocessing model. The preprocessing model will make sure each image goes through Gaussian filter, Clahe and resizing. The ADNI images will go through additional two steps which are skull stripping and slicing. Once the data is preprocessed the images will be split in training and testing data in 80:20 ratio. For the model training the training dataset will be provide to the model. In the model image features will be extracted through different models and post that it will go through the transfer learning models. Once the model is trained the images from the test dataset will be provided and the prediction will be done by model. The efficacy of the model will be judged on F1 score, accuracy, recall value and precision. The model will categorize the images in the three buckets as CN, AD & MCI.

### 3.2 Data preprocessing

The preprocessing approaches explored for identifying AD and MCI include noise reduction, CLAHE, Image resizing, and normalization. Noise reduction in MRI scans is achieved by using a Gaussian filter. This filter effectively reduces noise caused by different sources, better depicting the images for analysis. Gaussian filtering reduces intensity fluctuations and maintains structural information, enhancing MRI data quality (6). CLAHE improves the contrast of specific areas by adjusting the intensity levels according to local histograms. This leads to a more detailed representation of the essential structural features of AD research (7). Resizing an image to a defined dimension, such as 224×224 pixels, guarantees consistency and compatibility with CNN models. This process maintains the structural data of the image for analytical purposes. Normalization is a process that makes intensity levels similar across MRI scans. This helps in accurate and comparative analysis by guaranteeing that intensity distributions are the same (8).

The designated preprocessing techniques enhance the accuracy and comprehensibility of MRI data in identifying AD and MCI in both the ADNI and OASIS datasets. In addition, some prominent steps of preprocessing involved for the ADNI dataset:

Skull Striping: The U-Net architecture has an encoder-decoder structure incorporating skip links, similar to ResNets (10). Regarding skull stripping, the network takes a 3D MRI image as input and produces a binary mask that identifies the brain area. During learning, the network can divide the brain into distinct segments by predicting which pixels are part of the brain and which are not. The encoder component of the U-Net collects features from the input image at various scales, while the decoder component increases the resolution of these features to produce a segmentation mask that matches the resolution of the input image. Spatial information is using skip links that facilitate conserved and accurate localization. The neural network is taught using a dataset consisting of MRI images and their matching manually generated skull-stripped masks (11). After training, the U-Net may automatically perform skull stripping on newly acquired MRI images, making it a significant asset in neuroimaging research and clinical practice (9, 12).

Slicing: After removing the skull, slice the brain along the three axes (axial, coronal, and sagittal) to get cross-sectional slices. This procedure entails segmenting the three-dimensional (3D) pictures into two-dimensional (2D) slices, which record distinct brain structure viewpoints. After getting the slices, visually assess their quality. Select three slices for a better qualitative representation than the rejected ones. This reduces noise and highlights the most essential areas of MRI imaging (11).

**Figure 2:**
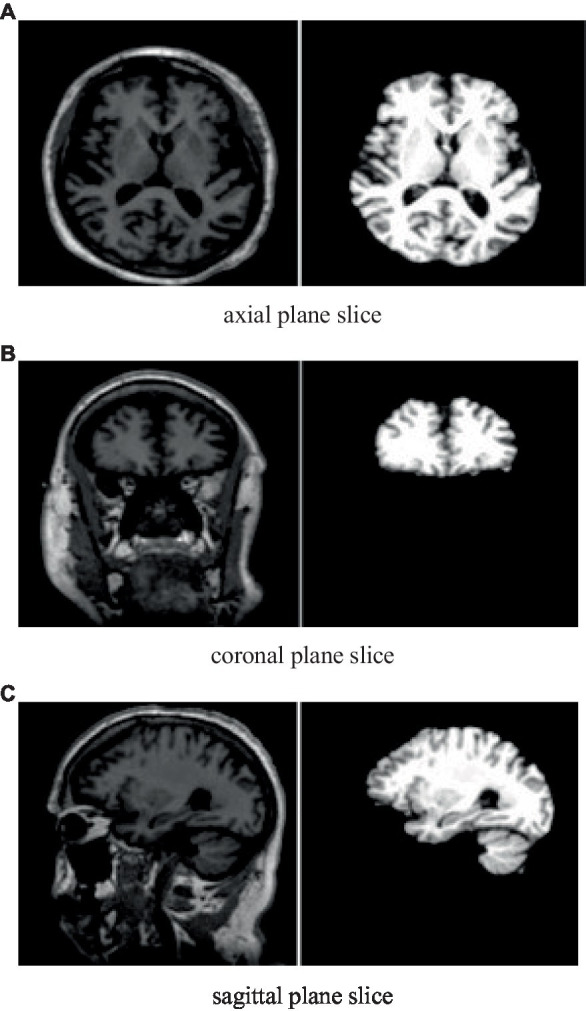
Slices of pre (Left) and post (Right) skull stripping (4). **(A)**: axial plane slice, **(B)**: Coronal plane slice, **(C)**: Sagittal plana Slice.

The images in Figure 2 show cross-sectional views before and after skull stripping, demonstrating the effects of the preprocessing method.

### 3.3 Prominent CNNs

ResNets: Residual Networks (ResNets) utilize shortcut connections between layers to facilitate residual learning. The residual learning approach entails acquiring knowledge about the residual mapping rather than the direct mapping of the input data, thereby enabling the efficient training of intense networks. Shortcut connections facilitate the propagation of gradients across layers and effectively address the disappearing gradients often seen in deep neural networks. ResNet comprises numerous residual blocks, including several convolutional layers and shortcut connections. This design enables the network to capture intricate input data aspects effectively (15).

**Figure 3:**
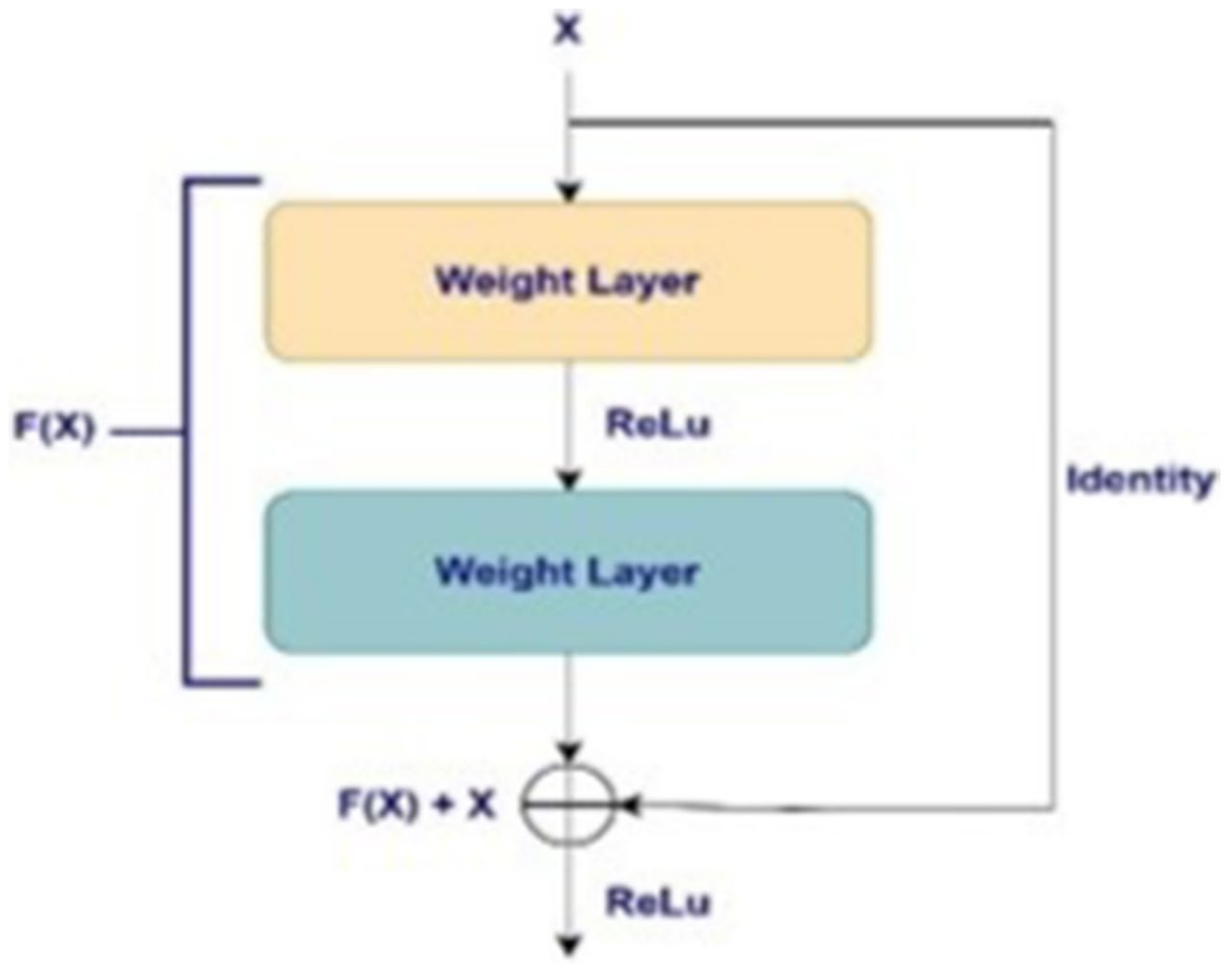
Shortcut connection used by ResNet.

ResNet can employ shortcut connections, bypassing one or more layers. The shortcut connections merely execute identity mapping; the results of these connections are aggregated with those of the layered layers. When many layers are appended, vanishing gradient issues frequently arise, preventing backpropagation from updating the weights of the initial layers. The problem might be remedied through the incorporation of an identity link. The ResNet architecture facilitates the direct propagation of gradients in the opposite direction, allowing them to traverse from the later layers to the initial filters via an identity link. By incorporating residual learning, the method improves the CNN architecture and renders it more applicable to the training of deep networks. A plain and simple network with a more significant number of layers tends to have more errors, but ResNet, which has specific layer configurations such as 50 and 101, has a superior capacity to handle deeper networks (15, 21, 22). Figure 3 presents the concept of shortcut connections.

ResNet familiarizes the conception of residual learning, where the layer transforms the input into a layer, and a shortcut connection bypasses one or more layers. Equation 1 presents the residual learning through Shortcut connection, understanding the basic building blocks of ResNets and how they are combined to form the architecture. This is expressed mathematically as:


(1) Output(Y)=F(Input(X))+Input(X)

Here, F(input(X)) represents the transformation performed by the layer.

A ResNet block typically consists of two convolutional layers followed by a shortcut connection. Let us denote input to the block as X, output as Y, and the residual function as F(X) (15). In Equation 2 the output y is computed as:


(2) $$Y=F\left(X,\left\{W_{i}\right\}\right)+X\;$$

where Wi are the weights of the convolutional layers.

ResNet has several layers, and these basic blocks are stacked together. The architecture consists of convolutional layers, batch normalization, ReLU activations, and residual blocks.

Let us consider a single convolutional layer within the residual function to simplify and derive this equation. Equation 3 calculates the output Y1 of the convolutional layer is given by:


(3) $$Y1=\sigma \left(W_{1}*X+b_{1}\right)\;$$

Here, W1 is the weights, b1 is the bias, σ is the activation function (commonly ReLU), and ∗ denotes convolution.

Now, let us consider another convolutional layer with output Y2 which can be calculated as Equation 4:


(4) $$Y2=\sigma \left(W_{2}*Y_{1}+b_{2}\right)\;$$

The residual function F(X) can be represented as the composition of these two layers:


(5) $$F\left(X\right)=\sigma \left(W_{2}*\left(\sigma \left(W_{1}*X+b_{1}\right)\right)+b_{2}\right)\;$$

Substituting the expression for F(X) of Equation 5 into the Equation 1, we get:


(6) $$Y=\sigma \left(W_{2}*\left(\sigma \left(W_{1}*X+b_{1}\right)\right)+b_{2}\right)+X\;$$

Equation 6 represents the forward pass through a single residual block.

The beauty of ResNet architecture lies in the ability to learn the identity mapping (i.e., Y = X) if needed. If the optimal transformation for a block is close to the identity mapping, the weights of the convolutional layers can be adjusted to approach the identity function, allowing for easier optimization during training (15, 23).

Each ResNet network consists of numerous convolutional layers, pooling layers, and fully connected layers with varying output sizes and numbers of filters. The advantages include improved accuracy with increased depth and overcoming the degradation problem observed in shallower networks. The disadvantages may include higher computational complexity, as indicated by the increase in floating-point operations (FLOPs), which measures the number of floating-point operations a neural network performs during inference or training with deeper networks (15).

ResNet-50: ResNet-50 uses residual learning to solve the degradation issue of deeper neural networks by creating skip connections or shortcuts that enable information to move directly across layers. The model consists of 50 layers, which include convolutional, pooling, and fully linked layers, using residual blocks as the fundamental components. Each residual block has many convolutional layers and a shortcut link to help the network learn abstract features (15).

ResNet-50 can train deeper networks without the vanishing gradient issue, improving performance on complex datasets. ResNet-50’s skip connections simplify training optimization, speeding convergence and improving generalization. ResNet-50 can be helpful for image classification and feature extraction because of its novel design and efficient training processes (18, 21).

ResNet-101: ResNet-101 is a CNN composed of precisely 101 layers. The construction of this architecture utilizes bottleneck blocks, which consist of three layers. It entails laying out various convolutional blocks with unique weights and additional elements, such as batch normalization and ReLU activations. The method employs residual learning to tackle degradation issues and enhance accuracy by leveraging higher depth. The network incorporates shortcut connections to facilitate residual learning, offering the choice between identity mapping or projection shortcuts. The model is trained using batch normalization, stochastic gradient descent (SGD), weight decay, and dropout. This approach achieves high accuracy and successfully addresses optimization challenges encountered in regular networks. ResNet-101 consists of recurring blocks with different filter quantities and other properties. The design guarantees that the number of parameters, depth, breadth, and computing cost remain identical to those of plain networks (15, 24).

ResNet-152: Its 152 layers make ResNet-152 one of the deepest convolutional neural networks. Multiple convolutional layers and identity mappings in residual blocks enable feature extraction at various abstraction levels. Skips in ResNet-152 let information flow directly from previous layers to subsequent ones, maintaining gradient flow and simplifying training optimization. Deep and skip connections improve this architecture’s image recognition performance, including accuracy, convergence during training, and the ability to handle vanishing gradient issues in deep neural networks (15, 24).

DenseNet-201: The Dense Convolutional Network (DenseNet) is characterized by a dense connection structure, which enables effective feature reuse and rapid model generation. The DenseNet-201 connects layers feed forwardly by utilizing feature maps from previous levels as inputs and producing feature maps for subsequent layers. The network has a total of a(a + 1)/2 direct connections for nodes, i.e., a, which successfully alleviates the vanishing-gradient problem, improves feature propagation, encourages feature reuse, and decreases parameter count. The architecture comprises many compact blocks, including convolutional layers alternated with transition layers, which reduce dimensionality and regulate the complexity of the model. This architectural design facilitates extracting features and propagating gradients, effectively tackling the issue of disappearing gradients in deep neural networks (13).

The main benefits of this approach are eliminating unnecessary features, less computational burden, and increased understanding of the model’s behavior due to dense connections. These advantages result in enhanced accuracy and efficiency while performing deep-learning tasks (11).

EfficientNet-B0: EfficientNet-B0 uses compound scaling to adjust the network’s depth, breadth, and resolution equally. This leads to the creation of smaller and more precise models. The fundamental idea is to attain the best possible performance within computing limitations by carefully managing the model’s depth, breadth, and resolution. The design incorporates inverted bottleneck blocks, squeeze-and-excitation blocks, and movable inverted bottleneck blocks, which optimize the use of parameters and processing resources. EfficientNet-B0 demonstrates exceptional performance, increased precision, reduced processing requirements, and adaptability, making it suitable for resource-limited settings such as mobile devices and edge computing (14).

### 3.4 Transfer learning

Transfer learning is a potent approach in ML that entails adjusting a pre-trained model from one task to another associated task, thereby capitalizing on the information acquired during the initial training. This strategy dramatically enhances the model’s performance while decreasing the need for extensive datasets in the target domain (25). Transfer learning allows researchers to optimize specific tasks by refining pre-existing CNN models such as ResNet-50, ResNet-101, ResNet-152, DenseNet-201, and EfficientNet-B0. These models have been extensively trained on datasets like ImageNet (11).

**Figure 4:**
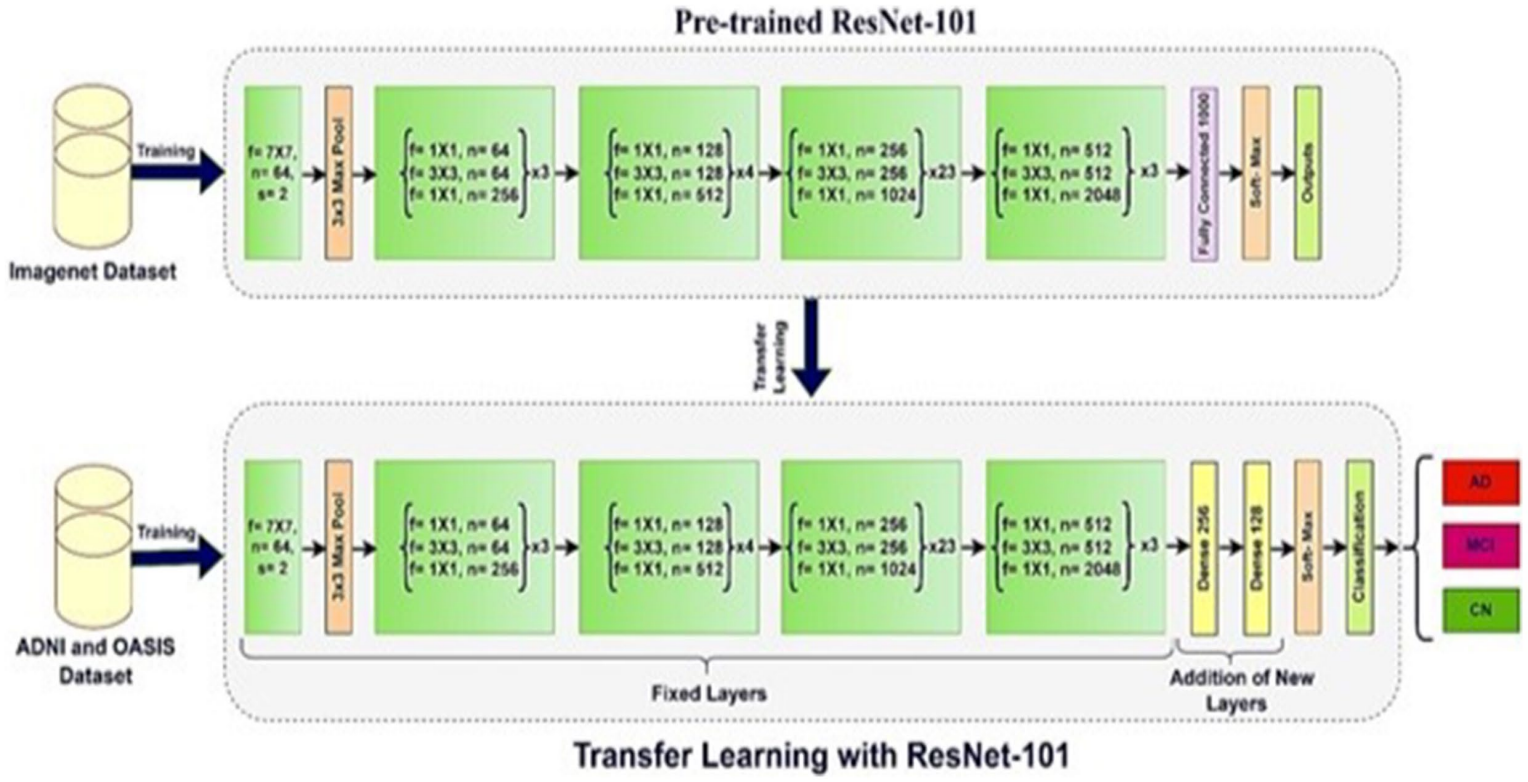
Transfer learning with ResNet-101.

Fine-tuning, an essential component of transfer learning, is modifying the model’s characteristics to match the patterns and correlations of the target problem. For example, ResNet and EfficientNet topologies sometimes improve by including extra dense layers, usually 256 and 128 units. This method allows models to specialize in activities beyond their initial training goals. Transfer learning allows models to use the information from pre-training on extensive datasets, enabling quicker convergence and enhanced generalization when fine-tuned on particular datasets. This strategy simplifies the process of developing models and improves performance in different applications (25, 26). The demonstration of the employment of transfer learning on ResNet-101 is presented in Figure 4, which removes the top layer and adds a new layer.

## 4 Results and discussion

The experiment was conducted utilizing a system including quadruple NVIDIA RTX A6000 GPUs, each equipped with 32 GB of memory, resulting in a combined processing capability of 194.8 TFLOPS. The system also included 64 GB of RAM and an AMD EPYC 7232P Octa Core CPU.

### 4.1 Dataset

The ADNI and OASIS datasets comprise human macroscopic MRI data, encompassing both individuals in good health and those who have received a diagnosis of Alzheimer’s and MCI disease. The ADNI and OASIS datasets employed in the investigation, renowned for their unrestricted access, provide researchers with invaluable resources for examining the human brain’s structural characteristics via MRI imaging. These datasets enable inquiries into both typical brain anatomy and pathological alterations that are linked to Alzheimer’s and MCI disease. The robust prediction model can be integrated with the MRI system so that it act as a helpful resource to the doctors.

ADNI: The ADNI dataset, a vast resource for AD progression research, uses MRI images to reveal deep brain anatomy. The ADNI longitudinal study uses MRI, PET, and other biological markers to identify biomarkers for early detection and tracking of AD. It allows in-depth analysis of brain area using bottom-to-top brain scanning axial visuals, with T1-weighted images improving anatomical structure analysis and problem detection. The ADNI incorporates several methods for participant and phantom scans. Participants undergo scanning utilizing a variety of sequences, including axial T2 STAR, axial 3D PASL, accelerated sagittal MPRAGE, sagittal 3D FLAIR, axial DTI, field mapping, axial rsfMRI with eyes open, and HighResHippocampus. The specific sequences may differ depending on the scanner’s manufacturer, for example, GE Systems for axial DTI scans and Philips Systems for resting-state fMRI and axial T2-FLAIR scans. This overview offers essential information on the imaging procedures and sequences used in the ADNI dataset. The dataset includes MRI scans from over 1,200 participants, each having multiple scans over time (4).

The age cohort-specific Alzheimer’s disease progression analysis is possible from 20 to 90. Further processing involved extracting 2D slices from the original T1-weighted MRI scans and a processed collection after skull stripping. The distribution of these slices across different anatomical planes was recorded as follows and given in Tables 1, 2.

**Table 1:** ADNI axial and coronal planes slices (4).

Class	Train	Test	Total
AD	4,980	1,244	6,224
MCI	4,162	1,040	5,202
CN	6,605	1,651	8,256

**Table 2:** ADNI sagittal planes slices (4).

Class	Train	Test	Total
AD	4,644	1,160	5,804
MCI	3,522	1,200	4,402
CN	3,912	978	4,890

This breakdown provides detailed insight into the composition of the dataset, which is crucial for understanding the distribution of data for training and testing purposes across different classes and anatomical planes (4). When working with MRI images many computational complexity’s need to be considered like image size and resolutions. Data augmentation need to be applied so that model has good number of images for the training. The preprocessing and feature extraction should be robust so that noise can be handled. Optimizing these factors is crucial for achieving efficient and effective analysis of MRI data in medical applications.

This investigation focused on three classes: CN, AD, and MCI, which had corresponding MRI scan counts of 159, 123, and 100, respectively. The dataset was partitioned into training and testing sets to facilitate deep learning tasks, wherein training comprised 80% of the data and testing included 20%. The CN group allocated 127 scans for training and 32 scans for testing, whereas the AD group utilized 99 scans for training and 24 scans for testing. For assessment purposes, there are 20 scans for testing of the MCI and 80 scans for training.

OASIS: The OASIS dataset is accessible to the public for investigation. It comprises cross-sectional MRI data from 416 people aged 18 to 96 years. Among these individuals, 100 have been diagnosed with AD at a very low to moderate stage. The dataset comprises T1-weighted MRI images for each participant, enabling a broad spectrum of analytical methodologies. The dataset has undergone de-identification, meticulous quality screening, and post-processing to provide standardized anatomical measurements. The inclusion of measures such as estimated total intracranial volume (eTIV) and normalized whole-brain volume (nWBV) offers valuable insights into the structural changes in the brain associated with aging and AD (5, 27). Table 3 shows the train-test (80–20%) split and quantity of MRI images for AD, MCI, and CN.

**Table 3:** OASIS class wise instances (27).

Class	Train	Test	Total
AD	390	98	488
MCI	4,800	1,200	6,000
CN	4,800	1,200	6,000

### 4.2 Performance metrics

Performance metrics quantify deep learning model performance. Many performance indicators include accuracy, precision, recall, and F1 score. Accuracy is the ratio of real positives and negatives to data points. Predicting the majority class may give the model high accuracy with imbalanced datasets, which may be misleading. The F1 score is a metric that combines recall and precision (26). On these metrics, precision, and recall calculations are predicated. Recall is the percentage of positive instances from the overall count of positive cases. At the same time, precision denotes the ratio of accurate optimistic predictions to the overall count of positive predictions. Incorporating false positives and false negatives, the F1 score is an exceptionally effective metric for assessing the performance of datasets containing unbalanced classes (28).

The specified CNN model employs the following Hyperparameters:

Although RMSprop is renowned for its capability to modify learning rates and manage sparse gradients, a learning rate of 0.02 may be excessively high and could be improved. The detection of AD and MCI are examples of multi-class classification tasks amenable to categorical cross-entropy. The batch size 64 frequently balances model stability and computational efficiency. Although 50 training epochs are a reasonable starting point, the validation loss must be closely monitored to prevent overfitting, and early halting should be considered.

### 4.3 Analysis

This investigation included three types of classification: first, multi-class classification in the AD vs. CN vs. MCI classes. The second is the Binary classification of AD and CN, and the third is MCI and CN. Comparing the predicted and observed labels yielded the accuracy of classification.

#### 4.3.1 Multi-class classification (AD vs. CN vs. MCI)

Table 4 compares employed CNNs onto the specified two datasets in the investigation of multi-class classification, i.e., AD, MCI, and CN. The outcomes presented in Table 4 demonstrate the model’s remarkable capacity to differentiate between cases of AD and MCI. The experimental results for the three-class classification experiment revealed that models become increasingly proficient in handling multi-class problems, as evidenced by their superior performance.

**Table 4:** AD vs. CN vs. MCI (multi-class classification).

CNN	Accuracy		Precision		Recall		F1 Score	
	A	O	A	O	A	O	A	O
ResNet-50	83.45	80.12	82.56	79.34	82.34	80.45	82.65	80.23
ResNet-101	98.21	97.45	94.67	93.12	94.89	93.67	94.78	93.45
ResNet-152	97.89	96.91	92.01	90.89	91.78	91.45	91.89	91.23
DenseNet-201	78.23	76.56	78.45	76.34	77.89	76.78	78.01	76.45
EfficientNet-B0	89.67	88.56	89.23	88.45	89.45	88.67	89.34	88.56

**Figure 5:**
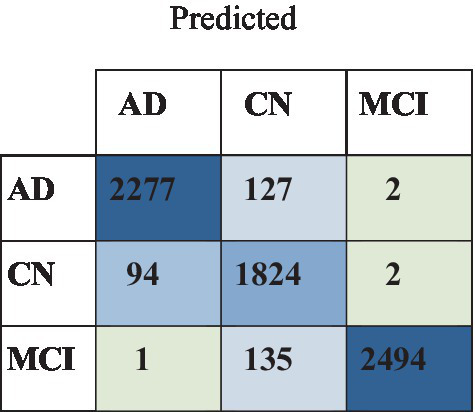
Confusion matrix of the ResNet-101 with ADNI dataset.

Quantification was performed on three cerebral components—white matter, gray matter, and cerebrospinal fluid—as part of the assessment of malady severity. The findings presented in Table 5 illustrate that the group comparing AD to CN to MCI attained exceptional levels of ResNet-101 Accuracy, Precision, Recall, and F1 Score. The findings show significant variations in performance across the CNN models in the multi-class classification test for AD, CN, and MCI. ResNet-101 scored the maximum accuracy and F1 score across both datasets, 98.21 and 94.78% for ADNI and 97.45 and 93.45% for OASIS, respectively, proving its ability to discriminate between the classes. ResNet-152 followed closely, achieving 97.89% accuracy for ADNI and 96.91% for OASIS. EfficientNet-B0, despite performing satisfactorily and scoring 89.67% for ADNI and 88.56% for OASIS. The F1 scores, which consider both accuracy and recall, reflected the patterns found in the individual measures, with ResNet-101 getting the most significant F1 scores for both datasets, followed by ResNet-152 and EfficientNet-B0. These findings indicate that ResNet-101 is the best model for this multi-class classification job, followed by ResNet-152, with EfficientNet-B0 trailing behind in performance. ResNet-101 has the highest accuracy level, meaning it can correctly put cases into each class. ResNet-152 and EfficientNet-B0 have lower accuracy measurements and fewer correct results. ResNet-101 did better than the others in memory to catch more true positives. ResNet-152 and EfficientNet-B0 had lower scores, which means they missed more false positives. Figure 5 illustrates the confusion matrix of the ResNet-101.

**Table 5:** Hyperparameters.

Sr No	Parameter	Value
1	Optimizer	RMSprop
2	Learning Rate	0.02
3	Loss Function	Categorical Cross-Entropy
4	Batch Size	64
5	Number of Epoch	20

#### 4.3.2 Binary classification (AD vs. CN)

**Figure 6:**
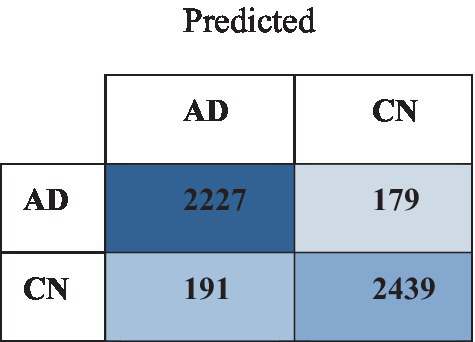
Confusion matrix of the ResNet-101 with ADNI dataset.

The outcomes of the binary classification test, distinguishing between AD and CN individuals, demonstrate diverse degrees of performance and are presented in Table 6. The AD versus CN group exhibited the subsequent categorization for the assessment primarily because of notable disparities in brain tissue region. The AD versus MCI group indices showed a reasonably high value but somewhat lower than the AD versus CN group. This observation aligns with predictions since MCI is pathologically more similar to AD than CN. Consequently, distinguishing between MCI and AD may be slightly more challenging. The findings indicated that the ResNet-101 model attained a notable level of accuracy in accurately categorizing the AD. The approach had a 92.34% accuracy in differentiating normal controls from AD patients. Again, ResNet-152 closely follows as the second-highest achiever, exhibiting robust performance across all criteria. DenseNet-201 has commendable performance but could be a lot better than ResNet-152. Conversely, EfficientNet-B0 has the least favorable performance compared to the other models. Figure 6 illustrates the confusion matrix of the ResNet-101 with the ADNI dataset.

**Table 6:** AD vs. CN (binary classification).

CNN	Accuracy		Precision		Recall		F1 Score	
	A	O	A	O	A	O	A	O
ResNet-50	87.45	85.32	86.78	88.23	86.23	89.45	86.78	87.89
ResNet-101	92.34	90.12	90.02	92.89	90.01	91.34	90.17	91.89
ResNet-152	89.67	88.23	88.78	90.45	88.34	91.23	88.78	89.67
DenseNet-201	85.67	84.12	84.78	86.23	84.45	87.12	84.78	85.67
EfficientNet-B0	78.23	77.45	77.78	79.56	77.89	80.12	77.78	78.23

#### 4.3.3 Binary classification (MCI vs. CN)

**Figure 7:**
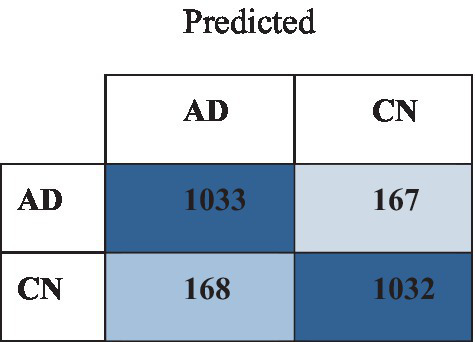
Confusion matrix of the ResNet-101 with OASIS dataset.

The outcomes of the binary classification of MCI and CN individuals demonstrate diverse degrees of performance and are presented in Table 7. In the endeavor of classifying MCI from CN, the efficacy of CNN models varied across metrics and datasets. ResNet-152 demonstrated the most exceptional overall performance among the assessed models, attaining an accuracy of 90.11% on the OASIS dataset and 89.56% on the ADNI dataset. Furthermore, the model exhibited high precision, recall, and F1 Score values across both datasets, signifying its resilient capability to differentiate between cases of MCI and CN. ResNet-101 demonstrated commendable performance. According to these findings, deeper CNN architectures, namely ResNet-152 and ResNet-101, exhibit notable efficacy in distinguishing between MCI and CN. Figure 7 illustrates the confusion matrix of the ResNet-152 with the OASIS dataset.

**Table 7:** MCI vs. CN (binary classification).

CNN	Accuracy		Precision		Recall		F1 Score	
	A	O	A	O	A	O	A	O
ResNet-50	75.23	69.45	80.34	74.56	75.34	68.23	78.45	71.23
ResNet-101	86.57	79.45	92.34	85.23	86.87	78.99	88.76	81.23
ResNet-152	89.56	90.11	93.12	86.12	87.34	79.45	89.23	82.34
DenseNet-201	84.32	77.89	90.45	82.67	84.45	76.78	87.34	79.56
EfficientNet-B0	82.45	76.23	89.12	81.34	82.56	74.93	85.12	78.32

*A,* ADNI; O, OASIS.

**Figure 8:**
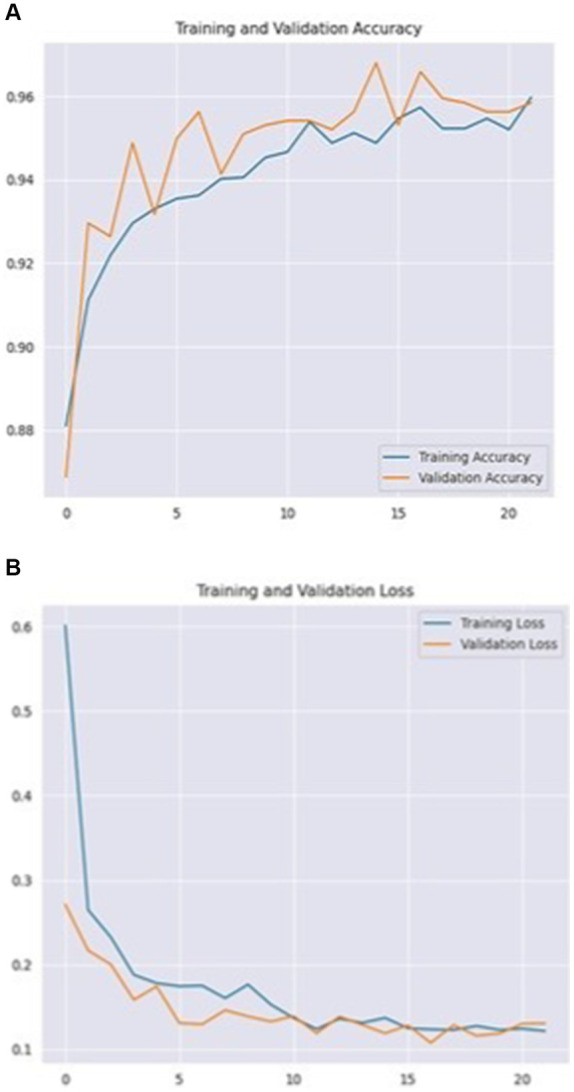
ResNet-101 training and validation accuracy **(A)** and training and validation loss **(B)** on multiclass classification.

The investigation found that ResNet-101 is best performed in the multi-classification and binary classification for the ADNI dataset; it is also well achieved with the OASIS dataset. The ResNet-101 model’s accuracy and loss were used to track and assess the training and validation process and presented through Figure 8. ResNet-101’s multi-class classification performance improves with time, as shown in Figures 8A,B, by showing training and validation accuracy and loss throughout epochs.

The training and validation accuracy consistently rises, while the training and validation loss consistently decreases, indicating that the model successfully integrates information from the training data.

### 4.4 Ablation study

The present investigation leverages the ADNI and OASIS datasets, which include comprehensive human macroscopic MRI data on healthy people and Alzheimer’s MCI patients. The ADNI dataset uses U-Net to remove the skull and brain, slicing along three axes for cross-sectional slices. These slices are quality-checked to minimize noise and highlight important MRI imaging areas. Prominent deep learning architectures like DenseNet-201, EfficientNet-B0, ResNet-50, ResNet-101, and ResNet-152 extract complex patterns to identify AD and MCI. The approach allows reliable ADNI and OASIS dataset processing, improving neurodegenerative condition comprehension and detection. The research examined how preprocessing techniques, deep learning architectures, and transfer learning methodologies affect the performance of models and compared their effectiveness. While Gaussian filters are frequently employed to reduce image noise, they might not be the most optimal approach to accentuate critical features in MRI data, especially when identifying Alzheimer’s and MCI. However, transfer learning is a highly effective method in deep learning, the results obtained from fine-tuning with specified pre-trained models and their effectiveness. While these models have been extensively trained on datasets like ImageNet, the performance of the transfer learning approach may have needed to be improved. The research examined the effects of incorporating dense layers of 256 and 128 units into each specified deep learning architecture after transfer learning from ImageNet-trained models to the ADNI and OASIS datasets. By comparing the efficacy of each architecture with and without additional layers, the research seeks to identify the structure that positively influences the distinction between CN, AD, and MCI groups the most. It can be deduced from the analysis that ResNet-101 exhibited the highest performance among the CNNs, with ResNet-152 following suit, whereas EfficientNet-B0 demonstrated the lowest performance. Across both datasets, ResNet-101 consistently attained the highest accuracy and F1 score, showcasing its efficacy in identifying AD and MCI. In the same way that ResNet-101 outperformed ResNet-152, albeit marginally, EfficientNet-B0 demonstrated subpar performance, suggesting limitations in its ability to classify data, particularly when coupled with transfer learning and preprocessing utilizing Gaussian filters.

Table 8 depicts an empirical comparison of AD and MCI identification using prominent deep-learning architectures, showing that our investigation achieved the maximum efficacy on both datasets.

**Table 8:** Comparative analysis.

# Classes	Deep learning architecture	Acc. (%)	Ref.
2	3D-CNN	93.00	(16)
2	EfficientNet-B2	93.30	(19)
3	ResNet18, AlexNet, SqueezeNet, VGG16, InceptionV3 & DenseNet	82.53	(21)
2	DenseNet	96.51	(30)
3	ResNet-18	69.10	(31)
3	VGG-16 & 19	95.35	(32)
3	DenseNet-201, EfficientNet-B0, ResNet-50, ResNet-101, and ResNet-152	98.21	Our

The analysis of deep learning architectures shows differing degrees of performance across various deep learning models. Table 8 presents the number of classes, the deep learning architecture used, and the attained accuracy. EfficientNet-B2 and 3D-CNN both demonstrated excellent accuracy in binary classification tests, suggesting their usefulness in the task. DenseNet demonstrated superior performance in a multi-class classification job with three classes, highlighting its resilience in addressing intricate classification challenges compared to other models. ResNet-18 obtained lower accuracy in a different multi-class classification scenario, indicating its shortcomings in hard classification tasks compared to other models. While in our investigation, the employed ResNet-101 obtained the highest accuracy in the multi-class classification challenge, showcasing enhanced performance. The findings emphasize the significance of choosing a suitable deep learning architecture according to the particular classification problem and the intricacy of the dataset. The research highlights the subtle variations in performance across different deep learning architectures, stressing the need to make well-informed choices to enhance model performance for specific tasks.

## 5 Conclusion

The investigation highlights the crucial use of modern neuroimaging and deep learning approaches in diagnosing and comprehending neurodegenerative disorders like Alzheimer’s and MCI. The extraction of valuable insights from complicated brain imaging using employed datasets ADNI and OASIS, which provide comprehensive MRI data, and implementation of advanced preprocessing techniques like skull stripping and segmentation on ADNI.

The U-Net architecture performed skull stripping on MRI images, successfully eliminating non-brain tissues. Specific deep learning models, such as DenseNet-201, EfficientNet-B0, ResNet-50, ResNet-101, and ResNet-152, were assessed for their ability to detect AD and MCI. Transfer learning is a powerful method for improving models, especially in situations with little datasets. Performance research shows that ResNet-101 regularly outperforms other models, followed closely by ResNet-152 with the datasets. ResNet-101 stands out as the best performer, attaining the most significant Accuracy levels and F1 Score on both datasets. This demonstrates its ability to effectively differentiate between people with AD, MCI, and CN instances, highlighting its resilience. ResNet-152 performed most in distinguishing between MCI and CN instances in a binary classification exercise with the OASIS dataset. The findings indicated that the CNN models performed well in this multi-class (29) and binary classification.

The study yielded promising results, yet several constraints and areas for future research remain to be addressed. Variations in model performance could stem from dataset characteristics and preprocessing methods. Further exploration of diverse preprocessing techniques and datasets is crucial to achieving a more comprehensive evaluation of model efficacy. While the research focused on a limited range of deep learning architectures, investigating additional structures and ensemble techniques may further enhance performance. Moreover, delving into model architecture choices and identifying biomarkers specific to Alzheimer’s and MCI could deepen our understanding of the underlying mechanisms of these disorders. Integrating clinical data with neuroimaging holds potential to improve diagnostic accuracy and prognostic predictions for Alzheimer’s and MCI. Future studies could benefit from combining these complementary sources of information to develop more robust and reliable predictive models. In the future research the research should focus on incorporating multimodality images like PET Scan with MRI for the more precise prediction and should try to use generative AI models on generating future brain images and use if for prediction.

## Data Availability

The original contributions presented in the study are included in the article/Supplementary material, further inquiries can be directed to the corresponding author.
